# Early life exposure to antibiotics and laxatives in relation to infantile atopic eczema

**DOI:** 10.1111/pai.13964

**Published:** 2023-05-26

**Authors:** Sarah El‐Heis, Sarah R. Crozier, Nicholas C. Harvey, Eugene Healy, Keith M. Godfrey

**Affiliations:** ^1^ Medical Research Council Lifecourse Epidemiology Centre University of Southampton Southampton UK; ^2^ NIHR Applied Research Collaboration Wessex Southampton Science Park, Innovation Centre Southampton UK; ^3^ NIHR Biomedical Research Centre University of Southampton and University Hospital Southampton NHS Foundation Trust Southampton UK; ^4^ Dermatopharmacology, Faculty of Medicine University of Southampton Southampton UK; ^5^ Developmental Sciences University of Southampton Southampton UK

**Keywords:** antibiotics, atopic eczema, laxatives, microbiome

**Abbreviations**
UK WPDC: UK Working Party Criteria for the Definition of Atopic Dermatitis
DAG: Directed Acyclic Graph
OR: odds ratio

## PEER REVIEW

The peer review history for this article is available at https://www.webofscience.com/api/gateway/wos/peer‐review/10.1111/pai.13964.

The risk of developing atopic eczema is influenced by various events pre‐conception, during pregnancy, and throughout the neonatal period.^1^, ^2^ Recent reports have suggested that early life exposure to microbiome altering medications, such as antibiotics and laxatives, could impact the risk of atopic eczema in infancy and childhood. For example, Lin et al., 2022, reported an increased risk of allergic disease in offspring whose mother used laxatives in pregnancy independent of laxative exposure in the offspring but no associations were found for maternal antibiotic use.^3^

As the evidence on this topic is sparse, we aimed to examine whether maternal gestational exposure to antibiotics or laxatives were associated with the risk of atopic eczema in infancy as well as the link between offspring antibiotic exposure in the first 12 months of life and risk of infantile atopic eczema.

The prospective Southampton Women's Survey^4^ mother–offspring cohort recruited 12,583 non‐pregnant women from the city of Southampton, UK. A total of 3158 who became pregnant were followed up during pregnancy; maternal gestational antibiotic and laxative exposure were contemporaneously recorded by research nurses during in‐person interviews in early (median 11.8 weeks' gestation) and late (median 34.5 weeks' gestation) pregnancy; *n* = 248 and *n* = 304 reported antibiotic exposure, and *n* = 67 and *n* = 72 laxative exposure in early and late pregnancy, respectively. At age 12 months, offspring antibiotic exposure was recorded (*n* = 2867, with 1433 exposed); of these, 10.1% were exposed to maternal antibiotic use in early pregnancy 12.3% to maternal antibiotic use in late pregnancy, 2.5% to maternal laxative use in early pregnancy and 3.4% to maternal laxative use in late pregnancy. Atopic eczema was ascertained using the UK Working Party Criteria (UK WPDC) for the Definition of Atopic Dermatitis^5^ at ages 6 (occurring at age 0–6 months) and 12 (occurring at age 6–12 months) months (cohort *n* = 2907 and 2870, respectively (*n* = 262 and 270, with eczema)).

**FIGURE 1:**
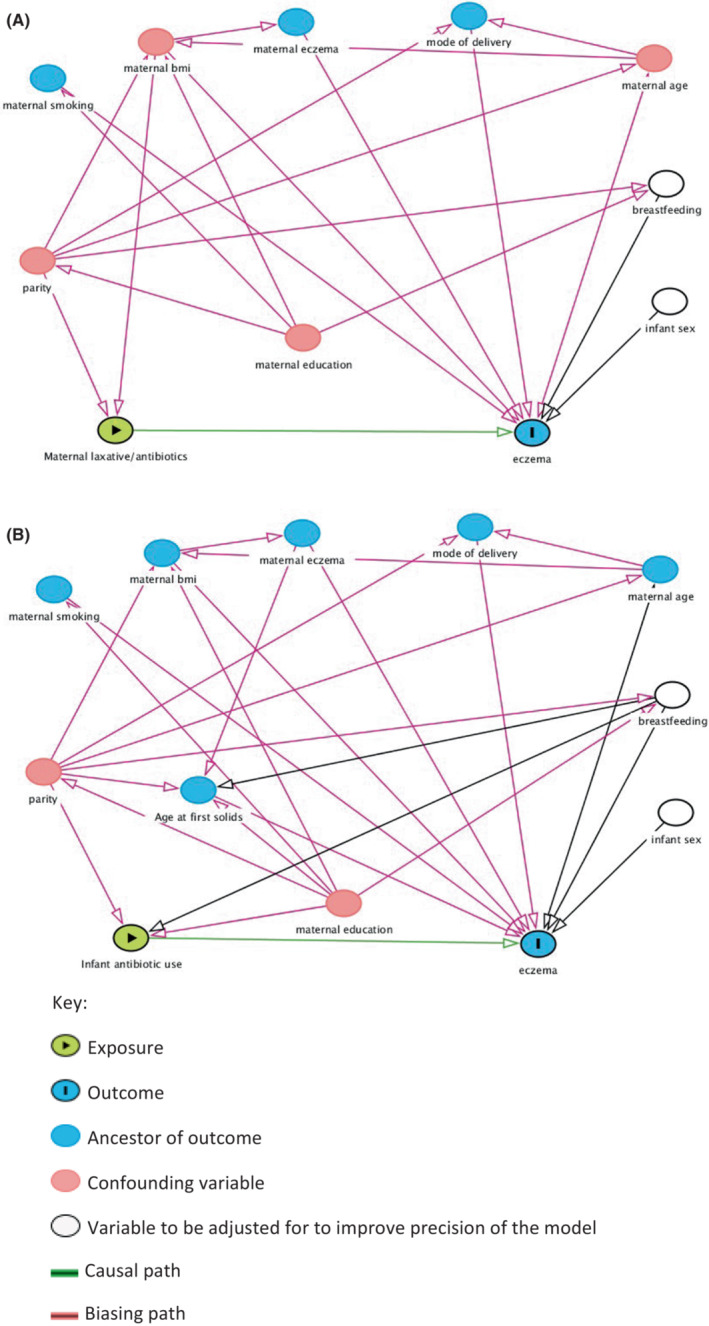
Directed Acyclic Graph (DAG) demonstrating the relationship between (A) maternal laxative and antibiotic use (exposure), infant atopic eczema (outcome). Confounders identified are: maternal BMI, parity, breastfeeding duration. Competing exposure identified is infant sex. (B) infant antibiotic use (exposure), and infant atopic eczema (outcome). Confounders identified are: maternal education, parity, breastfeeding duration. Competing exposure identified is infant sex.

We used Directed Acyclic Graphs (DAGs) to identify potential confounders and competing exposures that should be included in our statistical models to support causal interpretation.^6^ DAGitty v3.0, a web‐based application was used to create and analyze the DAG.^7^ The confounders in the DAG demonstrating the relationship between maternal laxative and antibiotic use (exposure), infant atopic eczema (outcome) are maternal BMI, parity, breastfeeding duration, and infant sex (Figure 1A), and in the DAG demonstrating the relationship between infant antibiotic use (exposure) and infant atopic eczema (outcome), confounders are maternal education, parity, breastfeeding duration, and infant sex (Figure 1B). Standard univariable and multivariable logistic regression analyses were carried out adjusting for confounders and competing exposures as identified by the DAGs to relate maternal use of antibiotics and laxatives in pregnancy, and infant antibiotic exposure to infantile atopic eczema at ages 6 and 12 months (Stata version 14.1, Statacorp LP). Antibiotic exposure captured at age 12 months was not used to predict infant eczema at 6 months.

We found no associations between maternal antibiotic use in pregnancy and infantile atopic eczema. Similarly, maternal laxative use had no associations with infantile atopic eczema at ages 6 or 12 months. Findings were unaltered when adjusting for confounders (Table 1). The use of antibiotics in infancy, however, was associated with an increased risk of atopic eczema at 12 months and remained significant in multivariable analyses, adjusted odds ratio (adjOR) (95% CI) 1.59 (1.23–2.07), *p* = .001 (Table 1). The risks of atopic eczema at age 12 months were similar in those who had antibiotics in the first 6 months and those who had antibiotics during age 6–12 months compared to those who did not have antibiotics. Exposure to antibiotics once only (*n* = 794), 2–3 times (*n* = 458), and more than three times (*n* = 164) in the first year of life were associated with similarly increased risks of atopic eczema, adjOR 1.51 (1.11–2.04), *p* = .008, 1.64 (1.15–2.33), *p* = .006 and 1.64 (0.97–2.7776), *p* = .6607, for once only, 2–3 times and more than three times, respectively.

**TABLE 1:** Atopic eczema at age 6 and 12 months in relation to exposure to maternal antibiotic use, maternal laxative use, and infant antibiotic use.

	Atopic eczema at age 6 months				Atopic eczema at age 12 months			
	*N*	adjOR	95% CI	*p* Value	*n*	adjOR	95% CI	*p* Value
Maternal antibiotic use^a^								
Early pregnancy	2532	0.87	0.70–1.08	.21	2467	0.97	0.80–1.18	.79
Late pregnancy	2401	1.06	0.89–1.25	.51	2336	1.10	0.93–1.30	.27
Maternal laxative use^a^								
Early pregnancy	2532	0.46	0.14–1.49	.20	2467	0.99	0.42–2.33	.98
Late pregnancy	2401	0.91	0.39–2.14	.83	2336	1.25	0.59–2.66	.56
Infant antibiotic use (in the first 12 months of life)^b^	NA^c^	NA^c^	NA^c^	NA^c^	2747	1.59	1.23–2.07	.001

^a^ Multivariable logistic regression analyses adjusting for maternal BMI, parity, breastfeeding duration, and infant sex.

^b^ Multivariable logistic regression analyses adjusting for maternal education, parity, breastfeeding duration, and infant sex.

^c^ This analysis was not undertaken as the outcome (atopic eczema at age 6 months) precedes the exposure (infant antibiotic use in the first 12 months of life).

The findings support evidence that postnatal antibiotic exposure is associated with the infant's risk of developing atopic eczema. Further work is needed to disentangle whether postnatal antibiotic exposure plays a causal role in infantile eczema, or whether antibiotic exposure occurs as a consequence of the eczema. Our data provide limited information on the condition for which antibiotics were prescribed and the sample size for documented individual infections is too small to draw conclusions (e.g., pneumonia 4%, bronchitis 15%), but the similar relationships for early and late infancy antibiotic exposure provide weak evidence against the antibiotics simply being prescribed as a consequence of the eczema.

Wohl et al reported an increased risk of atopic eczema at the age of 2 years in infants whose mothers took antibiotics during pregnancy.^8^ In our study, maternal gestational antibiotic or laxative exposure, however, were not related to infant atopic eczema. A systematic review of prenatal and infant antibiotic exposure and childhood allergic disease reported that prenatal antibiotics had an overall effect on eczema but the findings for infant antibiotic exposure were less consistent.^7^ Variation in definition of eczema or in study methodology may have contributed to this. In our study, atopic eczema was defined by UK WPDC which are recognized diagnostic criteria used in clinical and research settings and involved a standardized questionnaire and an examination undertaken by trained research nurses. Data on infant exposure to laxatives were not collected in this cohort, and the sample size in the pregnancy analyses was lower than that of the infant analyses as exposure data for some participants was not ascertained in early or late pregnancy, largely due to practical reasons. The DAGs used may have omitted some potential confounders (such as complementary feeding, for which links with eczema are contentious), but the approach is recognized as a robust method for identifying confounders in large observational epidemiological studies.^5^

Gut microbiome not only regulates the gut environment but it also influences the regulation of the microbiome of barrier sites such as the skin and the lungs. The timing of dysbiosis that is likely to impact the offspring's developing immune system is not known but it has been suggested that the first 6 months after birth should be considered a time of susceptibility as the microbiome develops rapidly and may induce long‐term immunological changes.^9^, ^10^ Supportive evidence from animal studies has shown that antibiotic exposure in neonatal mice was associated with shifts in the gut microbiome and subsequent signs of allergic asthma. These changes were not seen in exposed adult mice.^11^ Nonetheless, alterations in the microbiome may be related to atopic eczema and not a direct effect of antibiotics.

Maternal enteric dysbiosis may result from medications, which in turn may cause enteric dysbiosis of the fetus via the translocation of microbes through the bloodstream, and increase their predisposition to developing allergic disease. While we did not demonstrate evidence for this, we did identify that antibiotic use in infancy was associated with development of atopic eczema during the first 12 months of life. Our findings from a single observation have limited generalizability, but coupled with the results of recent studies we recommend further research to examine the impact of early life antibiotic and laxative exposure on the microbiome‐immune‐atopy axis.

## AUTHOR CONTRIBUTIONS

**Sarah El‐Heis:** Conceptualization; investigation; methodology; formal analysis; writing – review and editing; writing – original draft. **Sarah R. Crozier:** Methodology; formal analysis; writing – review and editing. **Nicholas C. Harvey:** Methodology; funding acquisition; writing – review and editing; supervision. **Eugene Healy:** Methodology; supervision; writing – review and editing. **Keith M. Godfrey:** Methodology; funding acquisition; investigation; supervision; writing – review and editing.

## FUNDING INFORMATION

This work was supported by grants from the Medical Research Council, British Heart Foundation, Food Standards Agency, Arthritis Research UK, National Osteoporosis Society, International Osteoporosis Foundation, Cohen Trust, European Union's Seventh Framework (FP7/2007–2013), projects EarlyNutrition and ODIN under grant agreement numbers 289346 and 613977, NIHR Southampton Biomedical Research Centre, University of Southampton and University Hospital Southampton NHS Foundation Trust, NIHR Musculoskeletal Biomedical Research Unit, University of Oxford, and British Lung Foundation. For the purpose of Open Access, the author has applied a Creative Commons Attribution (CC BY) license to any Author Accepted Manuscript version arising from this submission.

## CONFLICT OF INTEREST STATEMENT

KMG has received reimbursement for speaking at conferences sponsored by companies selling nutritional products. KMG is part of an academic consortium that has received research funding from Abbott Nutrition, Nestec, and Danone. CC reports personal fees from ABBH, Amgen, Eli Lilly, GSK, Medtronic, Merck, Novartis, Pfizer, Roche, Servier, and Takeda, outside the submitted work. NCH reports personal fees, consultancy, lecture fees and honoraria from Alliance for Better Bone Health, AMGEN, MSD, Eli Lilly, Servier, Shire, Radius Health, UCB, Consilient Healthcare, and Internis Pharma, outside the submitted work. The other authors have no competing interests.
